# Construction of a 5-feature gene model by support vector machine for classifying osteoporosis samples

**DOI:** 10.1080/21655979.2021.1971026

**Published:** 2021-10-08

**Authors:** Minwei Hu, Ling Zou, Jiong Lu, Zeyu Yang, Yinan Chen, Yaozeng Xu, Changhui Sun

**Affiliations:** a Department of Orthopedics, Ruijin Hospital LuWan Branch, School of Medicine, Shanghai Jiaotong University School of Medicine, Shanghai, China; b Department of Orthopedics, The First Affiliated Hospital of Soochow University, Suzhou, Jiangsu, China

**Keywords:** Osteoporosis, differentially expressed genes, protein–protein interaction, support vector machine, gene signature, bioinformatics

## Abstract

Osteoporosis is a progressive bone disease in the elderly and lacks an effective classification method of patients. This study constructed a gene signature for an accurate prediction and classification of osteoporosis patients. Three gene expression datasets of osteoporosis samples were acquired from the Gene Expression Omnibus database with pre-set criteria. Differentially expressed genes (DEGs) between normal and diseased osteoporosis samples were screened using Limma package in R language. Protein–protein interaction (PPI) network was established based on interaction data of the DEGs from the Human Protein Reference Database. Classification accuracy of the classifier was assessed with sensitivity, specificity and area under curve (AUC) using the pROC package in the R. Pathway enrichment analysis was performed on feature genes with clusterProfiler. A total of 310 differentially expressed genes between two samples were associated with positive regulation of protein secretion and cytokine secretion, neutrophil-mediated immunity, and neutrophil activation. PPI network of DEGs consisted of 12 genes. A SVM classifier based on five feature genes was developed to classify osteoporosis samples, showing a higher prediction accuracy and AUC for GSE35959, GSE62402, GSE13850, GSE56814, GSE56815 and GSE7429 datasets. A SVM classifier with a high accuracy was developed for predicting osteoporosis. The genes included may be the potential feature genes in osteoporosis development.**Abbreviations**DEGs: Differentially expressed genes; PPI: protein–protein interaction; WHO: World Health Organization; SVM: Support vector machine; GEO: Gene Expression Omnibus; KEGG: Kyoto Encyclopedia of Genes and Genomes; GO: Gene Ontology; BP: Biological Process; CC: Cellular Component; MF: Molecular Function; SVM: Support vector machines

## Introduction

Human skeletal system is in a process of constant renewal and dynamic balance, in which bone formation and bone resorption play important roles in maintaining the stability of the system [1]. Osteoporosis is a metabolic bone disease characterized by decreased bone content, destruction of bone microstructure and increased bone fragility. As a common clinical bone disease implicating about 200 million patients in the world, osteoporosis occurs when bone resorption exceeds bone formation [2]. According to the World Health Organization (WHO), the total number of osteoporosis patients in the world will reach 221 million by 2050. In China, osteoporosis is also one of the most frequently occurred diseases with an increasing incidence each year.

In recent years, machine learning methods have been increasingly applied to predict complex biological events. As a supervised machine-learning technique, support vector machine (SVM) is widely used in classification and pattern recognition. The SVM algorithm performs classification by establishing a multidimensional hyperplane that optimally distinguishes two classes through maximizing the margin between the two data clusters. The algorithm uses a special nonlinear function-kernel function to convert the input space into a multi-dimensional space, thereby obtaining a high discriminant ability [3]. SVM have also been used in medical applications [4–6]. SVM training algorithm builds a model to predict whether a new case falls into one of the categories by giving a set of training cases, and each training case is marked as belonging to one of two categories.

In this study, SVM was employed to classify osteoporosis and normal samples. Three data sets were used to construct and verify the prediction accuracy of the SVM classifier. The function and pathway information of the identified SVM classification feature genes was analyzed with a variety of bioinformatics methods to identify novel biomarkers.

## Materials and methods

### Data collection and processing

**Figure 1.**
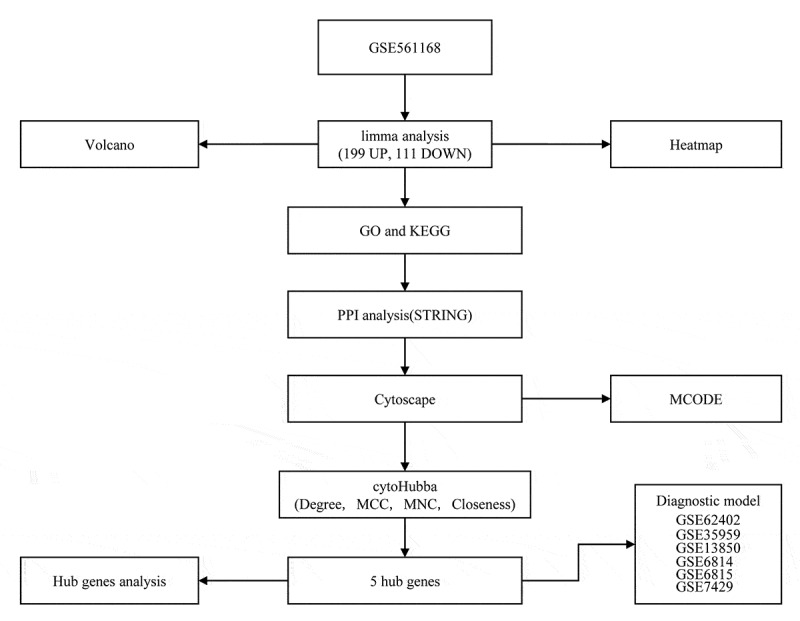
Work flow chart

The newest clinical follow-up data and RNA-seq data of osteoporosis cases were downloaded from Gene Expression Omnibus (GEO) to obtain gene expression profiles in the GSE56116, GSE62402, GSE35959, GSE13850, GSE56814, GSE56815 and GSE7429. GSE35959 dataset served as the training set, and GSE62402, GSE13850, GSE56814, GSE56815 and GSE7429 datasets served as validation datasets. For the chip data, probes were matched to genes, and those matched to multiple genes were removed, whereas multiple probes matching to the median of a gene was kept to acquire the gene expression profile. The sample data are all presented in Table 1. The workflow is presented in Figure 1.

**Table 1.** Sample information of datasets

Data set	Expression	Platforms
**GSE56116**		
Normal	3	GPL4133
Osteoporosis	10	
**GSE62402**		
High BMD	5	GPL5175
Low BMD	5	
**GSE35959**		
Normal	9	GPL570
Osteoporosis	5	
**GSE13850**		
High BMD	20	GPL96
Low BMD	20	
**GSE56814**		
High BMD	42	GPL5175
Low BMD	31	
**GSE56815**		
High BMD	40	GPL96
Low BMD	40	
**GSE7429**		
High BMD	10	GPL96
Low BMD	10

### Analysis of differentially expressed genes (DEGs)

Limma package was used to perform DEG analysis. Genes conforming to an adjusted p value of less than 0.05 and an absolute of fold change greater than 2 were defined as DEGs.

### Functional enrichment analysis

Genes with expression changes were annotated into Cellular Component (CC), Molecular Function (MF), and Biological Process (BP) of Gene Ontology (GO) and into Kyoto Encyclopedia of Genes and Genomes (KEGG) using the R software packages clusterProfiler (v 3.14.0) [7].

### Construction of PPI network for DEGs

We examined molecular interactions of the DEGs. A PPI network with mutant genes was generate using STRING database online (https://string-db.org/). The confidence score was set to 0.7 as the cutoff criteria, excluding disconnected nodes were hidden. Next, the interaction data in tsv format was downloaded to modify the PPI network in Degree, MNC, Closeness and MCC algorithms of Plug-in cytoHubba for Cytoscape (Version: 3.7.2) software in JAVA platform.

### Support vector machines (SVM) model

SVM, which is a supervised machine learning classification algorithm, determines sample type through estimating the degree of a sample belonging to a certain class [8]. For the GSE35959 training set, a SVM classifier was constructed based on the hub gene set using SVM method with the R package e1071 [9]. The performance of the SVM classifier was separately evaluated in the training set and 2 validation sets (GSE62402 and GSE7158).

## Results

A total of 310 differentially expressed genes between two samples were found to be associated with positive regulation of protein secretion and cytokine secretion, neutrophil-mediated immunity, and neutrophil activation. PPI network of DEGs consisted of 12 genes. A SVM classifier based on 5 feature genes was developed to classify different osteoporosis samples, showing a higher prediction accuracy and AUC for GSE35959, GSE62402, GSE13850, GSE56814, GSE56815 and GSE7429 datasets.

### Screening and functional analysis of DGEs

**Figure 2.**
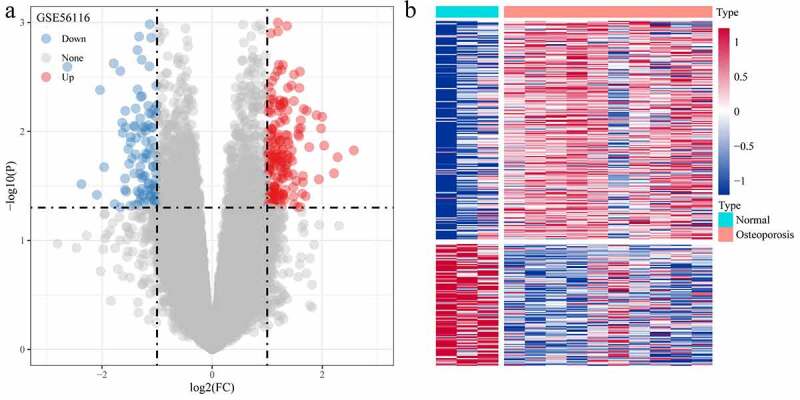
Screening of differentially expressed genes. (a) Volcano plot of differentially expressed genes in dataset GSE56116; (b) Heat map of differentially expressed genes

The DEGs between Normal and GSE56116 genotypes were calculated using the Limma package. The analytical results showed that there were 310 DEGs (111 down-regulated genes and 199 up-regulated genes) (Figure 2(a,b)).

**Figure 3.**
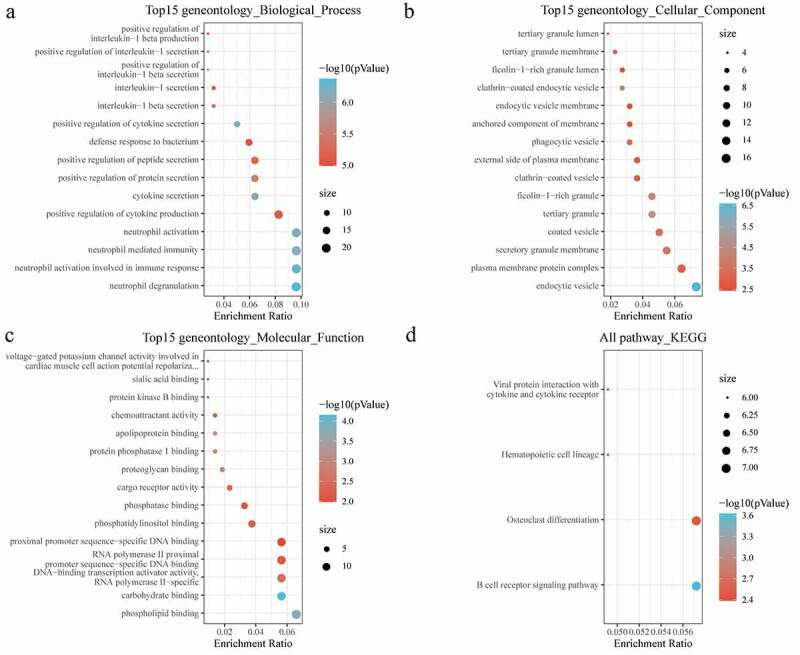
Functional enrichment of differentially expressed genes. (a) BP annotation of differentially expressed genes; (b) CC annotation of differentially expressed genes; (c) MF annotation of differentially expressed genes; (d) KEGG annotation of differentially expressed genes

Next, the biological function of DEGs was further analyzed with conducting GO and KEGG analysis. For biological process (BP) of GO analysis, the targeted genes were noticeably enriched to positive regulation of cytokine and protein secretion, neutrophil activation and neutrophil mediated immunity (Figure 3(a)). For the cellular component (CC), there were 47 pathways obviously enriched to these genes (Figure 3(b)). For molecular function (MF), genes were found to be closely related to phospholipid binding and DNA binding pathways (Figure 3(c)). Moreover, KEGG analysis showed that the DEGs were enriched to essential pathways associated with osteoporosis progression, including B-cell receptor signaling pathway, hematopoietic cell lineage, osteoclast differentiation, and viral protein interaction with cytokine and cytokine receptor (Figure 3(d)).

### Protein–protein interaction analysis

**Figure 4.**
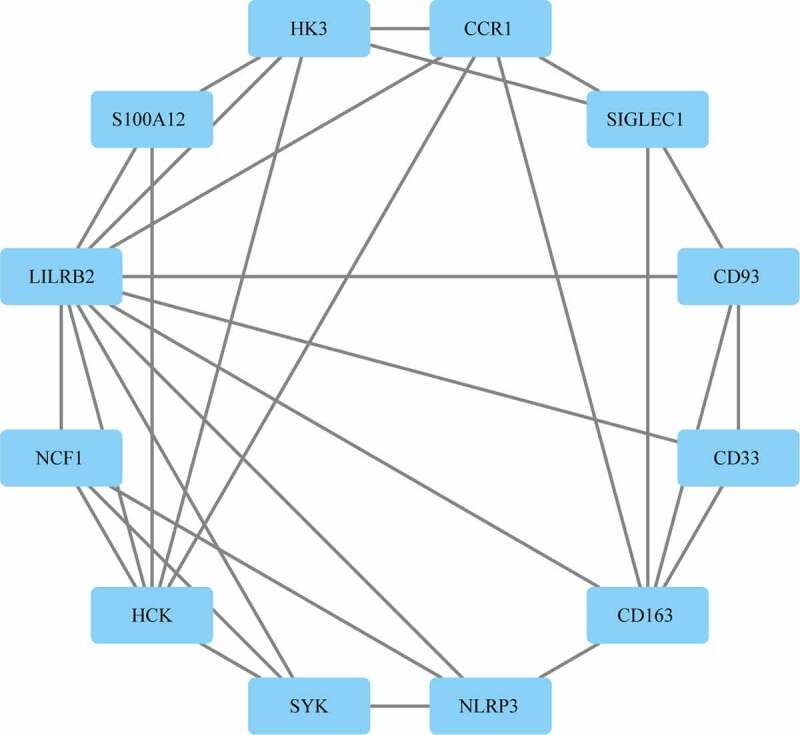
PPI analysis of the gene of the functional module

Protein–protein interaction (PPI) network analysis on a total of 310 DEGs was performed using STRING. According to Cytoscape 3.7.2 and its plug-in, 12 out of the 310 target genes were filtered by the target genes PPI network complex (MCODE1(MCODE.csv)) (Figure 4).

**Figure 5.**
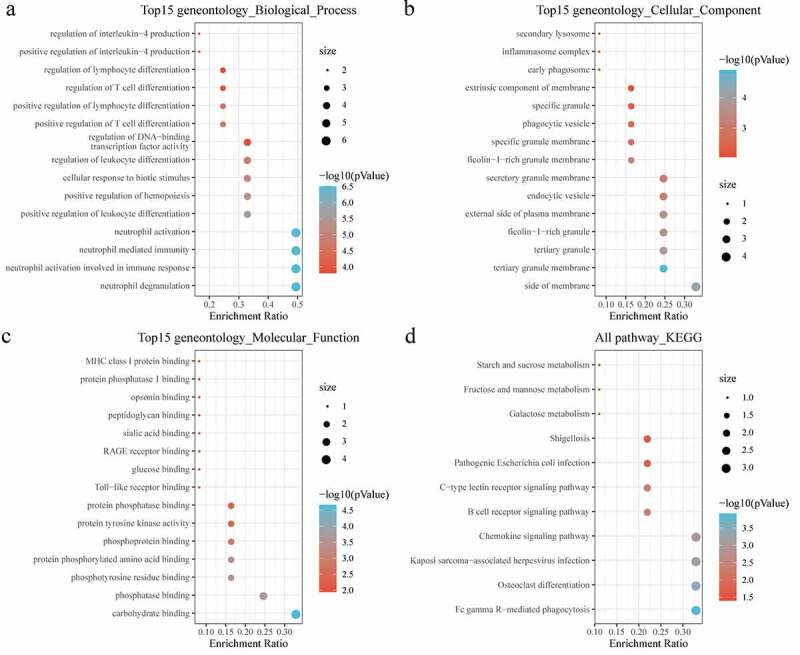
Functional enrichment of functional module genes. (a) BP annotation of functional module genes; (b) CC annotation of functional module genes; (c) MF annotation of functional module genes; (d) KEGG annotation of functional module genes

Furthermore, KEGG pathway analysis and GO functional enrichment analysis were conducted on 12 genes of MCODE1 module using R software package clusterProfiler (V 3.14.0). For BP of GO analysis, the targeted genes were found to be highly enriched to neutrophil-mediated immunity, neutrophil activation involved in immune response, positive regulation of leukocyte differentiation, regulation of lymphocyte differentiation (Figure 5(a)). For the CC, there were 24 pathways obviously enriched to these genes (Figure 5(b)). For the MF, there were 33 pathways noticeably enriched to these genes (Figure 5(c)). For KEGG analysis, the DEGs were enriched to essential pathways, such as chemokine signaling pathway, B-cell receptor signaling pathway, osteoclast differentiation (Figure 5(d)).

### Identification of hub genes

**Figure 6.**
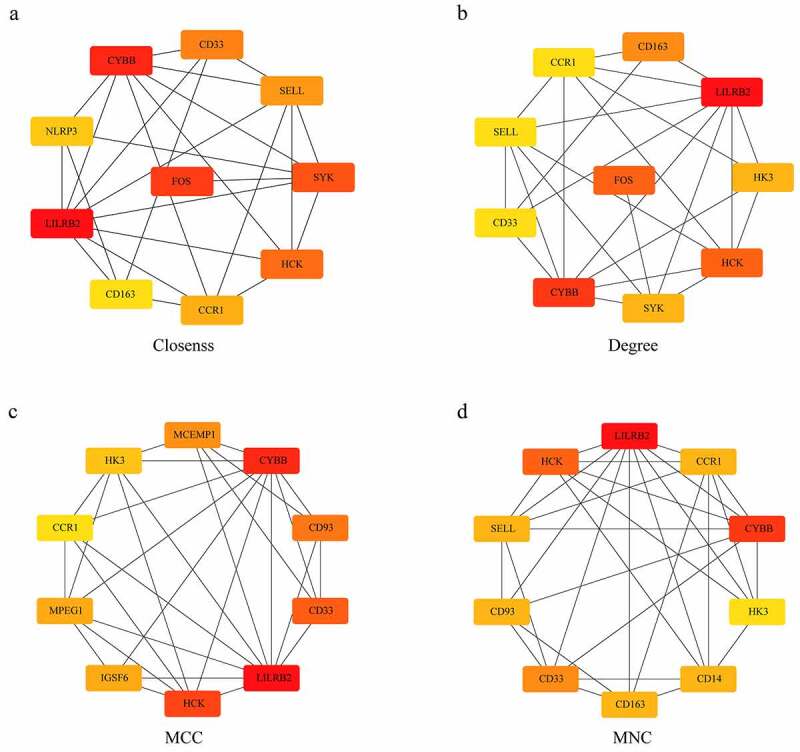
Identification of Hub genes. (a) PPI network diagram of hub genes obtained by Closeness algorithm. (b) PPI network diagram of hub genes obtained by MCC algorithm. (c) PPI network diagram of hub genes obtained by MNC algorithm. (d) PPI network of hub genes obtained by Degree algorithm

**Figure 7.**
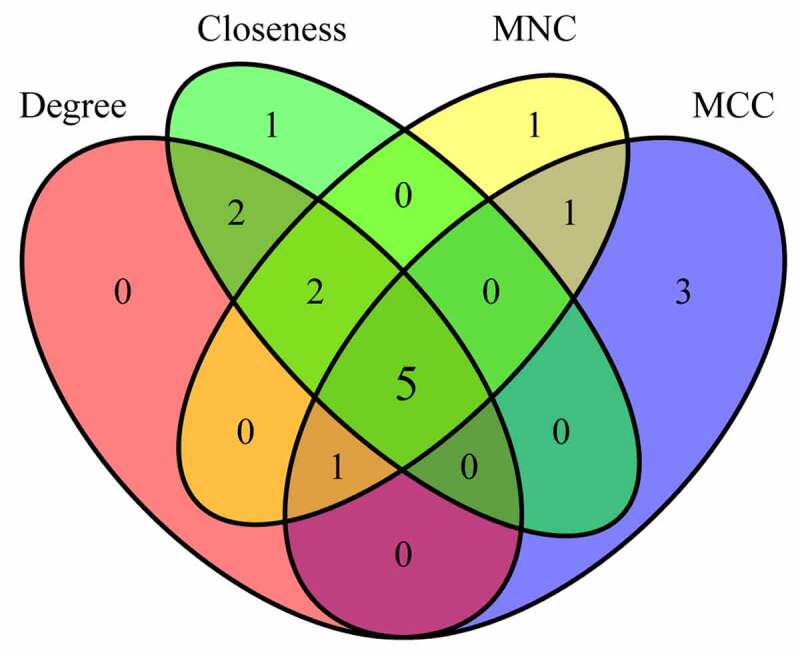
Venn diagram of hub genes identification

The degree, MNC, Closeness, and MCC algorithms of cytoHubba plug-ins for Cytoscape 3.7.2 software were applied to calculate and construct PPI networks based on 310 DEGs (Figure 6). Then, the genes obtained by these four algorithms were intersected, and we obtained five genes, which were CCR1, CD33, HCK, LILRB2 and CYBB (Figure 7). These five genes were regarded as final hub genes.

### Development and verification of the diagnostic model

**Figure 8.**
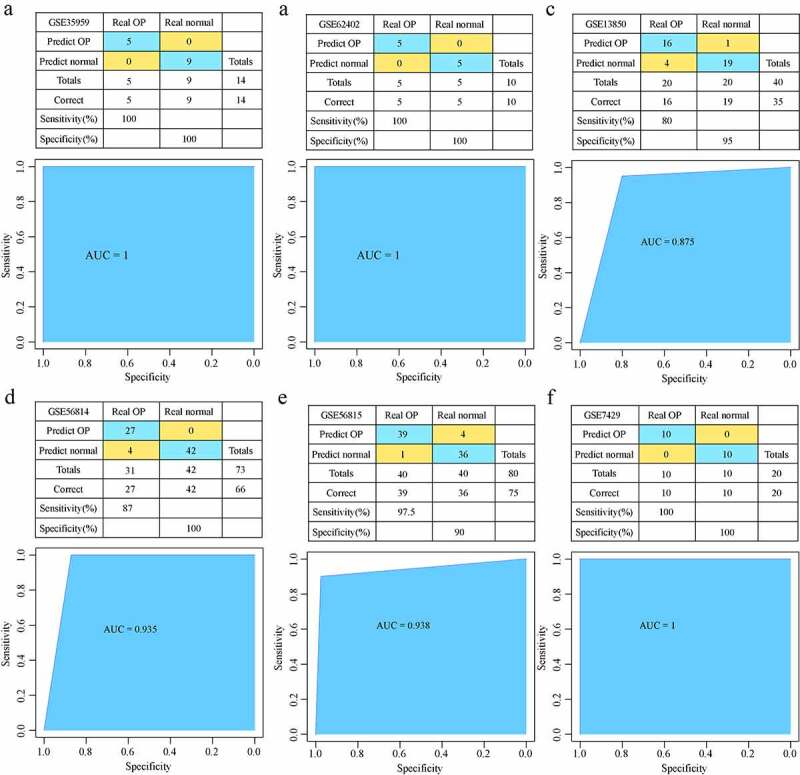
Development and verification of the model. (a) The classification result and ROC curve of the GSE35959 dataset sample by the diagnostic model; (b) The classification result and ROC curve of the GSE62402 dataset sample by the diagnostic model. (c) The classification result and ROC curve of the GSE13850 dataset sample by the diagnostic model; (d) The classification result and ROC curve of the GSE56814 dataset sample by the diagnostic model; € The classification result and ROC curve of the GSE56815 dataset sample by the diagnostic model; (f) The classification result and ROC curve of the GSE7429 dataset sample by the diagnostic model

Five hub genes from GSE35959 training data set were used to develop an expression spectrum and a SVM classification model. We found that the sample classification accuracy of the training data set was 100%, and that the sensitivity and specificity of the model were 100%, with an area under ROC curve (AUC) of 1 (Figure 8(a)). In the GSE62402 data set, similarly, all the 10 samples were correctly classified, showing a 100% classification sensitivity, specificity, accuracy of the model, with an area under ROC curve of 1 (Figure 8(b)). GSE13850 data set was used for verification, 35 of the 40 samples were correctly classified, the classification accuracy was as high as 87.5%, the model sensitivity was 80%, the specificity was 95%, and the area under the ROC curve was 0.875 (Figure 8(c)). In GSE56814 dataset, 66 out of 73 samples were correctly classified, the classification accuracy was as high as 94.5%, the model sensitivity was 87%, the specificity was 100%, and the area under the ROC curve was 0.935 (Figure 8(d)). In GSE56815 dataset, 75 of the 80 samples were correctly classified, the classification accuracy was as high as 93.8%, the sensitivity of the model was 97.5%, the specificity was 90%, and the area under the ROC curve was 0.938 (Figure 8(e)). In GSE7429 verification dataset, and 20 out of 20 samples were correctly classified, the classification accuracy was as high as 100%, the sensitivity and specificity of the model were 100%, and the area under the ROC curve was 1 (Figure 8(f)). These results indicated that the SVM classification model could accurately distinguish osteoporosis samples from normal samples; moreover, these five genes were reliable biomarkers for the diagnosis of osteoporosis.

## Discussion

Compared with traditional machine learning algorithms, SVM algorithm could greatly simplify computational complexity, as it applies a nonlinear mapping of the inner product kernel function to a high-dimensional space, and this is more suitable for classification using high-dimensional data but with few training queues in the selection of all available functions [8]. Studies demonstrated that using only one biomarker will undoubtedly reduce the accuracy of predicting disease prognosis [10–12]. Also, the generalization ability of SVM and advanced algorithm of artificial intelligence (AI) is better than neural networks in classifying small samples and is less prone to overfitting when combined with penalty terms. Considering the limited research samples, we applied SVM rather than deep learning for model development [13].

Most of the previous studies focused on the study of a single gene in Osteoporosis, such as Circular RNA Circ_0005564, or Circular RNA circ_0000020 promotes osteogenic differentiation via ceRNA mechanism [14,15]. At present, multi-gene combined diagnosis is also one of the research hotspots. After performing integrated microarray analysis, we screened 310 DEGs between osteoporosis patients and normal controls. Furthermore, gene biomarkers for osteoporosis were determined by Cytohubba, a plug-in for Cytoscape (Version: 3.7.2) software. A 5-gene combination (CCR1, CD33, HCK, LILRB2 and CYBB) was established as an optimal and effective biomarker for osteoporosis using SVM with feature selection and classification procedures. Moreover, the 5-gene classification model was 100% accurate in distinguishing normal patients from osteoporosis, showing 100% specificity and 90% sensitivity in three datasets.

Among the five genes, CCR1 is a major receptor for CCL3 (MIP-1α), which is a pro-inflammatory cytokine that stimulate osteoclasts activity and induces osteoclastogenesis [16–18]. To the best of our knowledge, the association between osteoporosis and the other four genes (CD33, HCK, LILRB2 and CYBB) was the first described in this study. CD33 is associated with a number of diseases, including acute leukemia and acute promyelocytic leukemia. Upon binding of ligands such as C1q or sulfonylated glycoproteins, two immunoreceptor tyrosine-based inhibitory motifs (ITIM) located in cytoplasmic tail of CD33 are phosphorylated by Src-like kinases such as LCK [19,20]. HCK is a member of the Src family of tyrosine kinases, and it mediates the degranulation and activation of NADPH oxidase during phagocytosis, mobilization of secretory lysosomes, resulting in a respiratory burst [21–24]. As a member of the leukocyte immunoglobulin-like receptor (LIR) family, LILRB2 could recognize HLA-G in complex with B2M/β-2 microglobulin and ninhydrin self-peptide (peptide-bound HLA-G-B2M), subsequently triggering the differentiation of myeloid suppressor cells and type 1 regulatory T cells. Both of the two could help actively sustain maternal-fetal tolerance [25–27]. Under the mutations in CYBB, NADPH oxidase could not assemble or function normally, and phagocytes will not be able to produce reactive oxygen species to kill foreign invaders, thereby leading to the dysregulation of neutrophil activity [28–31]. However, further research should be conducted to further explore the roles of those genes in osteoporosis.

## Conclusions

In summary, five genes significantly associated with osteoporosis were identified in this study, providing new understanding of the molecular mechanism of osteoporosis. These five genes were potential biomarkers for osteoporosis. But a lack of biological validation with a larger sample size was considered to be a limitation of this research. Future studies should also verify the diagnostic performance of the current gene model before clinical use.

## Data Availability

The data that support the findings of this study are openly available in GSE35959 [https://www.ncbi.nlm.nih.gov/geo/query/acc.cgi?acc=GSE35959], GSE62402 [https://www.ncbi.nlm.nih.gov/geo/query/acc.cgi], GSE13850 [https://www.ncbi.nlm.nih.gov/geo/query/acc.cgi], GSE56814 [https://www.ncbi.nlm.nih.gov/geo/query/acc.cgi], GSE56815 [https://www.ncbi.nlm.nih.gov/geo/query/acc.cgi], and GSE7429 [https://www.ncbi.nlm.nih.gov/geo/query/acc.cgi] datasets.

## Research highlights

- A protein interaction network was developed with bioinformatics for osteoporosis.
- CCR1, CD33, HCK, LILRB2, and CYBB were key genes in osteoporosis.
- A 5-gene diagnostic model was validated by support vector mechanism.
